# Increased Ih Current Is Associated with Reduced Hippocampal CA1 Excitability in a Mouse Model of Multiple Sclerosis

**DOI:** 10.1523/ENEURO.0160-26.2026

**Published:** 2026-07-07

**Authors:** Justin Read, Shaona Acharjee, Camila Pasquini, Gustavo R. Morel, Quentin J. Pittman, Carlos D. Gomez

**Affiliations:** ^1^Hotchkiss Brain Institute, University of Calgary, Calgary, Alberta T2N 4N1, Canada; ^2^Faculty of Medicine, Nursing and Health Sciences, School of Translational Medicine, Monash University, Melbourne, Victoria 3004, Australia; ^3^Instituto de Investigaciones Bioquímicas de La Plata "Prof. Doctor Rodolfo R. Brenner" (INIBIOLP), Facultad de Ciencias Médicas, Universidad Nacional de la Plata, La Plata, BA CP 1900, Argentina; ^4^Snyder Institute for Chronic Disease, Alberta Children’s Hospital Research Institute, Department of Physiology and Pharmacology, Cumming School of Medicine, University of Calgary, Calgary, Alberta T2N 4N1, Canada

**Keywords:** CA1 pyramidal neurons, experimental autoimmune encephalomyelitis, HCN channels, hyperpolarization-activated current, input resistance, intrinsic excitability

## Abstract

Multiple sclerosis (MS) is a chronic inflammatory disease characterized by demyelination and neurodegeneration, often accompanied by cognitive and emotional impairments. To investigate neuronal alterations associated with neuropsychiatric disorders, we investigated intrinsic excitability and synaptic properties of hippocampal CA1 pyramidal neurons in experimental autoimmune encephalomyelitis (EAE), a mouse model of MS. Whole-cell patch–clamp recordings were performed in acute hippocampal slices from presymptomatic and symptomatic female EAE mice and complete Freund's adjuvant-treated controls. Intrinsic properties were significantly altered in symptomatic EAE mice, with a reduction in firing frequency associated with ∼24% decrease in input resistance (Rin). However, action potential threshold, rheobase, and amplitude remained unaltered. Synaptic transmission was also preserved, as spontaneous excitatory and inhibitory postsynaptic current frequency and amplitude, along with paired-pulse ratio, showed no significant differences across groups. Importantly, we found that hyperpolarization-activated (Ih) currents, key regulators of neuronal excitability that have been implicated in neuropsychiatric disorders, were enhanced in symptomatic EAE mice, correlating with the observed decreased Rin. Pharmacological isolation of Ih currents using the blocker ZD7288 confirmed this increase. Western blots from the EAE hippocampal tissue revealed elevated HCN1 expression, a major Ih-conducting channel subunit in CA1 pyramidal neurons, in symptomatic EAE. Together, these findings indicate that upregulated Ih is associated with reduced Rin and may contribute to limiting neuronal excitability in the hippocampus of symptomatic EAE, independent of changes in excitatory and inhibitory synaptic transmission. This intrinsic adaptation may contribute to hippocampal dysfunction associated with MS, highlighting Ih modulation as a potential therapeutic target.

## Significance Statement

Cognitive problems are common in people with multiple sclerosis (MS), but the underlying brain mechanisms remain unclear. The hippocampus, a region critical for learning and memory, is especially vulnerable to MS-related damage. Here we show that, in a mouse model of MS, hippocampal neurons become less able to fire electrical signals, even though their synaptic connections remain intact. This reduced excitability was linked to changes in the neurons' own electrical properties rather than their inputs. These findings highlight an overlooked mechanism of hippocampal dysfunction in MS and point to new directions for protecting memory and cognition.

## Introduction

Multiple sclerosis (MS) is a chronic autoimmune disorder of the CNS characterized by inflammatory demyelination, neurodegeneration, and profound cognitive, sensory, and motor impairments (Lassmann, 2018). The disease affects millions worldwide, significantly impacting the quality of life of affected individuals. Traditionally, MS research has focused on the immune-mediated proteolysis of myelin and subsequent axonal damage; however, growing evidence suggests that intrinsic neuronal dysfunction also contributes to disease pathophysiology (Dutta and Trapp, 2011; Woo et al., 2024). In particular, alterations in neuronal excitability may play a key role in the cognitive impairments observed in MS patients, yet the mechanisms underlying these changes remain poorly understood.

The hippocampus, a brain region essential for learning and memory, is highly susceptible to MS-related pathology. Structural imaging has demonstrated significant hippocampal atrophy in MS patients, which correlates with cognitive decline (Sicotte et al., 2008; Planche et al., 2017; Cortese et al., 2024). In several brain regions of MS patients or EAE mice, there are reports of reduced synaptic excitation, disrupted inhibitory control, and changes in ion channel properties (Dutta et al., 2011; Nistico et al., 2013; Di Filippo et al., 2015; Acharjee et al., 2018; Cortese et al., 2024). Together, these findings indicate that multiple aspects of neuronal function may be altered in MS, highlighting the importance of understanding these changes for the development of strategies to preserve cognitive function.

Experimental autoimmune encephalomyelitis (EAE) is the most widely used animal model of MS, recapitulating key pathological features such as inflammatory demyelination, gliosis, and neurodegeneration (Ransohoff, 2012; Lassmann and Bradl, 2017). In addition to enabling study of these classic hallmarks, EAE models have provided important insights into neuronal dysfunction associated with MS. While previous studies have focused on synaptic alterations, few have systematically examined changes in intrinsic excitability in hippocampal neurons during disease progression.

In this study, we investigated the intrinsic and synaptic properties of hippocampal CA1 pyramidal neurons in EAE mice to determine how neuronal excitability is altered by autoimmune inflammation. Using whole-cell patch–clamp electrophysiology, we examined action potential (AP) properties, input resistance, synaptic transmission, and hyperpolarization-activated currents (Ih) in presymptomatic and symptomatic EAE mice, as well as control [complete Freund's adjuvant (CFA)-treated] animals. We focused on Ih currents, a key regulator of neuronal excitability mediated by hyperpolarization-activated cyclic nucleotide-gated (HCN) channels, with HCN1 being a major contributor to Ih in CA1 pyramidal neurons (Robinson and Siegelbaum, 2003). Previous studies have shown that alterations in Ih can modulate neuronal firing and input resistance, potentially contributing to compensatory mechanisms that limit excitability (George et al., 2009; He et al., 2014).

Our findings reveal that symptomatic EAE mice exhibit a significant reduction in the firing rate of CA1 pyramidal neurons, driven by decreased input resistance, while AP properties and synaptic transmission remain largely unaffected. Importantly, we identified a robust increase in Ih current and HCN1 protein expression in symptomatic EAE mice, suggesting that increased Ih activity contributes to altered intrinsic membrane properties and reduced excitability. These findings are consistent with the possibility that Ih upregulation represents an adaptive response to neuroinflammatory conditions. By identifying alterations in Ih signaling associated with hippocampal intrinsic dysfunction in EAE, our study provides insight into cellular mechanisms that may contribute to cognitive dysfunction in MS.

## Materials and Methods

### EAE induction in mice

All procedures were approved by the University of Calgary Animal Care Committee and conducted in accordance with institutional and national guidelines. Female mice were used throughout the study, reflecting the higher prevalence of MS in females compared with males (3:1 ratio; Constantinescu et al., 2011). EAE was induced in 8- to 10-week-old female C57BL/6N mice (Charles River Laboratories), housed under specific pathogen-free conditions (four per cage, 12 h light/dark cycle). Mice received a subcutaneous immunization with 100 µg of myelin oligodendrocyte glycoprotein peptide (MOG_35–55_; AnaSpec) emulsified in CFA containing 4 mg/ml heat-killed *Mycobacterium tuberculosis* (Difco Laboratories). Pertussis toxin (200 ng; List Biological Laboratories) in sterile PBS was administered intravenously at the time of immunization and again 48 h later, as previously described (Ousman et al., 2007). Control animals received CFA and pertussis toxin without MOG peptide and are referred to as “CFA” mice throughout the text.

Mice were randomly assigned to the EAE or CFA group to ensure balanced cohort sizes. No anesthesia was used for the immunization protocol. Mice were monitored daily and scored for neurological deficits based on a standard five-point EAE clinical scale (Acharjee et al., 2018). Presymptomatic animal groups were killed 8 d postimmunization and verified to have no detectable motor deficits, which typically begin after 10–13 d postimmunization. Symptomatic EAE mice were selected based on clinical scores of 2–3 corresponding to tail and hindlimb paralysis, 17 d postimmunization. All experiments were conducted during the light phase of the circadian cycle.

### In vitro hippocampal electrophysiology

To investigate intrinsic and synaptic properties of CA1 pyramidal neurons, we performed whole-cell patch–clamp recordings in acute hippocampal slices prepared from control (CFA), presymptomatic, and symptomatic EAE mice. Animals were deeply anesthetized with isoflurane and transcardially perfused with an ice-cold slicing solution containing the following (in mM): 87 NaCl, 2.5 KCl, 25 NaHCO_3_, 0.5 CaCl_2_, 7 MgCl_2_, 1.25 NaH_2_PO_4_, 25 d-glucose, and 75 sucrose. Both slicing and artificial cerebrospinal fluid (aCSF; see below) were continuously bubbled with 95% O_2_ and 5% CO_2_ to maintain pH at 7.4. Brains were rapidly removed, and 300-µm-thick transverse slices of the dorsal hippocampus were obtained at a 45° angle from the coronal plane using a VT1200S vibratome (Leica), as previously described (Gomez et al., 2019; Gomez et al., 2021). We specifically targeted the dorsal hippocampus due to established functional and biophysical differences along the septotemporal axis (Strange et al., 2014).

Slices were incubated in aCSF (in mM: 126 NaCl, 2.5 KCl, 26 NaHCO_3_, 2.5 CaCl_2_, 1.5 MgCl_2_, 1.25 NaH_2_PO_4_, and 10 D-glucose) at 32°C for 60 min and then held at room temperature for an additional 30 min before recordings. For recordings, slices were continuously perfused with oxygenated aCSF at 32°C at a rate of 1–2 ml/min. CA1 pyramidal neurons were visually identified under differential interference contrast and infrared microscopy by their location in the pyramidal layer and somatic morphology. To record intrinsic membrane properties, pipettes were filled with a potassium-based internal solution containing the following (in mM): 108 K-gluconate, 8 Na-gluconate, 2 MgCl_2_, 8 KCl, 1 EGTA, 4 K_2_-ATP, 0.3 Na-GTP, and 10 HEPES. The solution was adjusted to pH 7.2 with KOH and to an osmolarity of ∼285 mOsm. To record synaptic transmission, a cesium-based internal solution was used to block potassium currents, composed of the following (in mM): 108 d-gluconic acid, 108 CsOH, 5 TEA-Cl, 2.8 NaCl, 20 HEPES, 0.4 EGTA, 4 MgATP, 0.3 NaGTP, 10 phosphocreatine Na_2_, and 1 QX-314. The pH was adjusted to 7.2 with CsOH and the osmolarity to ∼305 mOsm. Liquid junction potentials were compensated, and only cells with a stable series resistance (<20 MΩ) that did not change by >20% throughout the experiment were included in the analysis.

Cells were allowed to stabilize for ≥5 min before recording resting membrane potential (RMP), defined as the membrane voltage with no injected current. Input resistance (Rin) was determined as the slope of the linear portion of a voltage-current plot constructed from the steady-state voltage response to step current injections from RMP ranging from −100 up to 50 pA in 30 pA increments and 500 ms duration. Firing frequency was assessed by injecting depolarizing steps (80–320 pA, 500 ms) from RMP. AP threshold and rheobase were determined using ramp current injections (increments of 50 pA over 2 s); threshold was defined as the voltage at which the rate of rise (dV/dt) exceeded 10 mV/ms. AP amplitude was measured from the first spike. To investigate Ih currents, two protocols were employed. In the first, inward currents were recorded in response to a series of 250 ms hyperpolarizing voltage steps (from −70 to −130 mV in 10 mV increments) from a holding potential of −70 mV. In the second protocol, cells were held at −40 mV and subjected to a series of 750 ms voltage steps from −140 to −40 mV in 20 mV increments. Here, tetrodotoxin (1 μM) was added in the bath to prevent AP generation and rebound firing during hyperpolarizing protocols, allowing accurate isolation of Ih. To isolate the hyperpolarization-activated current (Ih), the selective Ih channel blocker ZD7288 (10 µM; Sigma-Aldrich) was bath-applied, and current subtraction was performed. Changes in input resistance in the presence or absence of ZD7288 were also assessed to estimate the contribution of Ih to membrane conductance. During ZD7288 application, membrane potential was maintained near the initial resting level by injecting holding current when necessary before measuring input resistance.

For synaptic recordings, cells were voltage-clamped at −70 mV to record spontaneous excitatory postsynaptic currents (sEPSCs), which were isolated in the presence of picrotoxin (100 µM) to block GABA_A_-mediated inhibition. For inhibitory synaptic recordings, cells were voltage-clamped at 0 mV to record spontaneous inhibitory postsynaptic currents (sIPSCs) in standard aCSF. sEPSC and sIPSC frequency and amplitude were analyzed offline using MiniAnalysis (Synaptosoft) using thresholds of five times RMS noise levels. Evoked EPSCs (eEPSCs) were elicited every 10 s via bipolar concentric tungsten electrodes (SNEX-100, David Kopf Instruments) placed in the stratum radiatum to stimulate Schaffer collaterals at ∼50% of maximal response intensity. A 50 ms interstimulus interval was used to assess paired-pulse ratio (PPR), calculated as the amplitude of the second peak relative to the first (P2/P1). eIPSCs were recorded using the same stimulation paradigm, and IPSC PPR was calculated similarly. Signals were acquired at 10 kHz and low-pass filtered at 1 kHz during acquisition using a Multiclamp 700B amplifier (Molecular Devices), digitized with a Digidata 1322A interface, and recorded using pClamp 9.2 (Molecular Devices). Data were analyzed using Clampfit 9.2 and MiniAnalysis.

### Protein extraction and immunoblotting

To ensure consistency with the electrophysiological experiments, the hippocampal tissue was collected using the same dissection approach described above, and the CA1 region was microdissected and snap-frozen on dry ice. The hippocampal tissue from control (CFA) and symptomatic EAE mice was then homogenized in ice-cold RIPA buffer supplemented with protease and phosphatase inhibitors using a TissueLyser LT (Qiagen). Lysates were centrifuged at 14,000 × *g* for 10 min at 4°C to remove insoluble material, and the supernatant was retained. Protein concentrations were determined using a BCA protein assay (Thermo Fisher Scientific). Equal amounts of protein (20 µg per lane) were separated by SDS–PAGE on 10% TGX Stain-Free acrylamide gels (Bio-Rad Laboratories), activated using the ChemiDoc MP Imaging System (Bio-Rad Laboratories), and transferred to 0.44 µm Immobilon-P PVDF membranes (MilliporeSigma). Following transfer, total protein load was visualized using stain-free technology (Bio-Rad Laboratories) and used for normalization.

Membranes were blocked for 1 h in 3% BSA in Tris-buffered saline containing 0.1% Tween 20 (TBST) and then incubated overnight at 4°C with a primary antibody anti-HCN1 (dilution 1:1,000 in 3% BSA/TBST, N70/28, #AB_ 2115181, UC Davis/NIH NeuroMab Facility). After washing, membranes were reprobed with 1:10,000 rabbit anti-mouse-HRP (48–370-H, Antibodies Incorporated) for 1 h at room temperature. Immunoreactive bands were visualized using enhanced chemiluminescence (ECL substrate, Thermo Fisher Scientific) and imaged using the ChemiDoc MP system. Band intensity corresponding to HCN1 was quantified using Image Lab Software v6.0 (Bio-Rad Laboratories) and normalized to the total protein content of each lane.

### Data analysis

All data analyses were performed using GraphPad Prism version 10.0 (GraphPad Software). Comparisons of intrinsic membrane properties, synaptic parameters, and Western blot data were performed using either unpaired Student's *t* tests or one-way or two-way ANOVA followed by a post hoc test for comparisons. All results are expressed as mean ± SEM. For electrophysiological experiments, statistical tests were conducted on the number of recorded cells (*n*), but numbers of animals (*N*) are also indicated. For molecular data, analyses were based on number of animals. Statistical significance was set at *p* < 0.05.

## Results

### Clinical progression of EAE and definition of experimental stages

**Figure 1.**
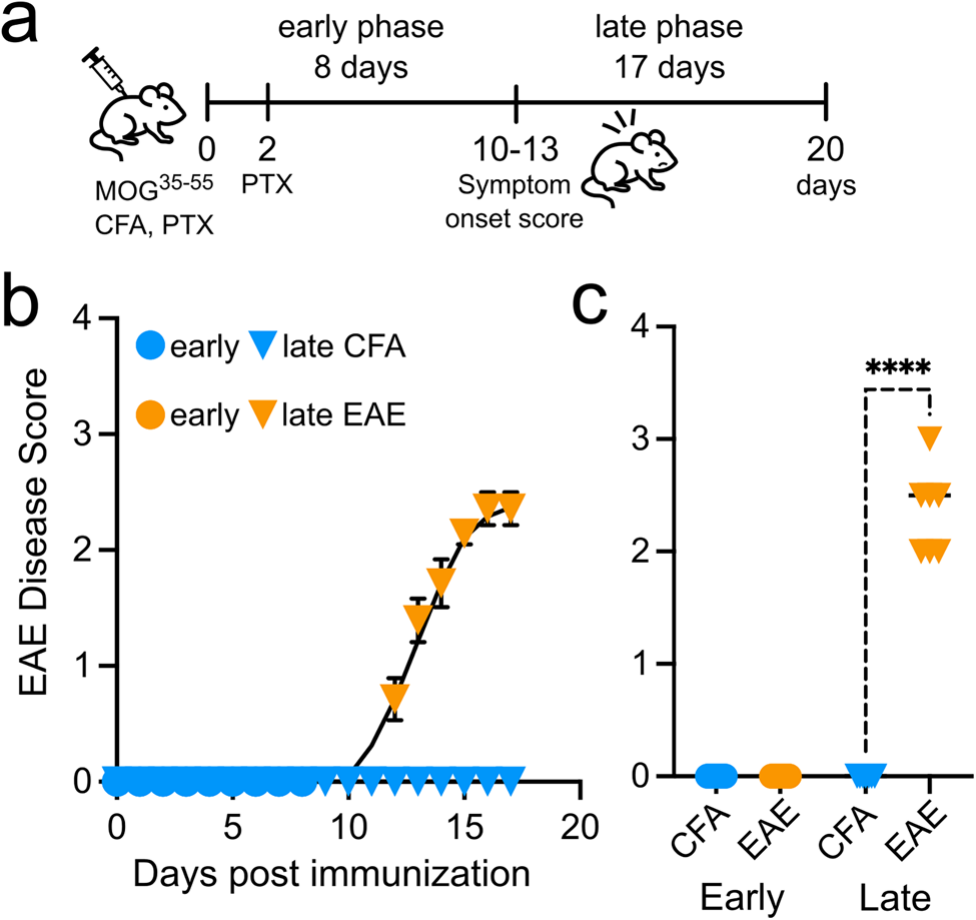
Clinical progression and staging of EAE. ***a***, Schematic representation of the EAE induction protocol and experimental timeline. Mice received subcutaneous injections of MOG^35–55^ emulsified in CFA and intravenous injections of pertussis toxin (PTX) on Day 0; PTX was injected again 48 h later. Based on clinical evaluation, an early phase (8 d postimmunization) and a late phase (17 d postimmunization) were defined. ***b***, Daily clinical scores for CFA-treated control mice and EAE mice. CFA-injected controls remained asymptomatic throughout the experimental period. EAE mice developed neurological symptoms beginning ∼10–13 d postimmunization, followed by progressive worsening. Circles indicate early phase and inverted triangles indicate late phase. ***c***, Summary of clinical scores during early and late phases for CFA and EAE groups. Late EAE mice exhibited significantly higher clinical scores compared with CFA controls (****p < 0.0001). Data are shown as mean ± SEM.

To monitor disease progression and animal welfare, EAE mice were scored daily for neurological symptoms using a standard clinical scoring scale (Fig. 1). Following immunization with MOG^35–55^, animals developed neurological deficits after a symptom-free period, with disease onset occurring ∼10–13 d postimmunization. Based on clinical scores, we defined an early phase of the disease (8 d postimmunization), during which mice displayed no detectable neurological impairment, and a late phase (17 d postimmunization), characterized by clear motor deficits (Fig. 1*b*).

Consistent with this classification, EAE mice exhibited a progressive increase in disease score during the late phase, whereas CFA-treated control mice remained asymptomatic throughout the experimental period (Fig. 1*b*). Quantification of clinical scores confirmed a significant increase (one-way ANOVA test, *F*_(3,23)_ = 157.7; *p* < 0.0001) in disease severity in late-stage EAE mice compared with CFA controls (CFA 0, *N* = 7; EAE 2.357 ± 0.1429; *N* = 7; *p* < 0.0001; Fig. 1*c*), while no differences were observed between CFA and EAE mice during the early phase (*p* > 0.9999).

### Altered intrinsic electrophysiological properties of CA1 pyramidal neurons in symptomatic but not presymptomatic EAE mice

**Figure 2.**
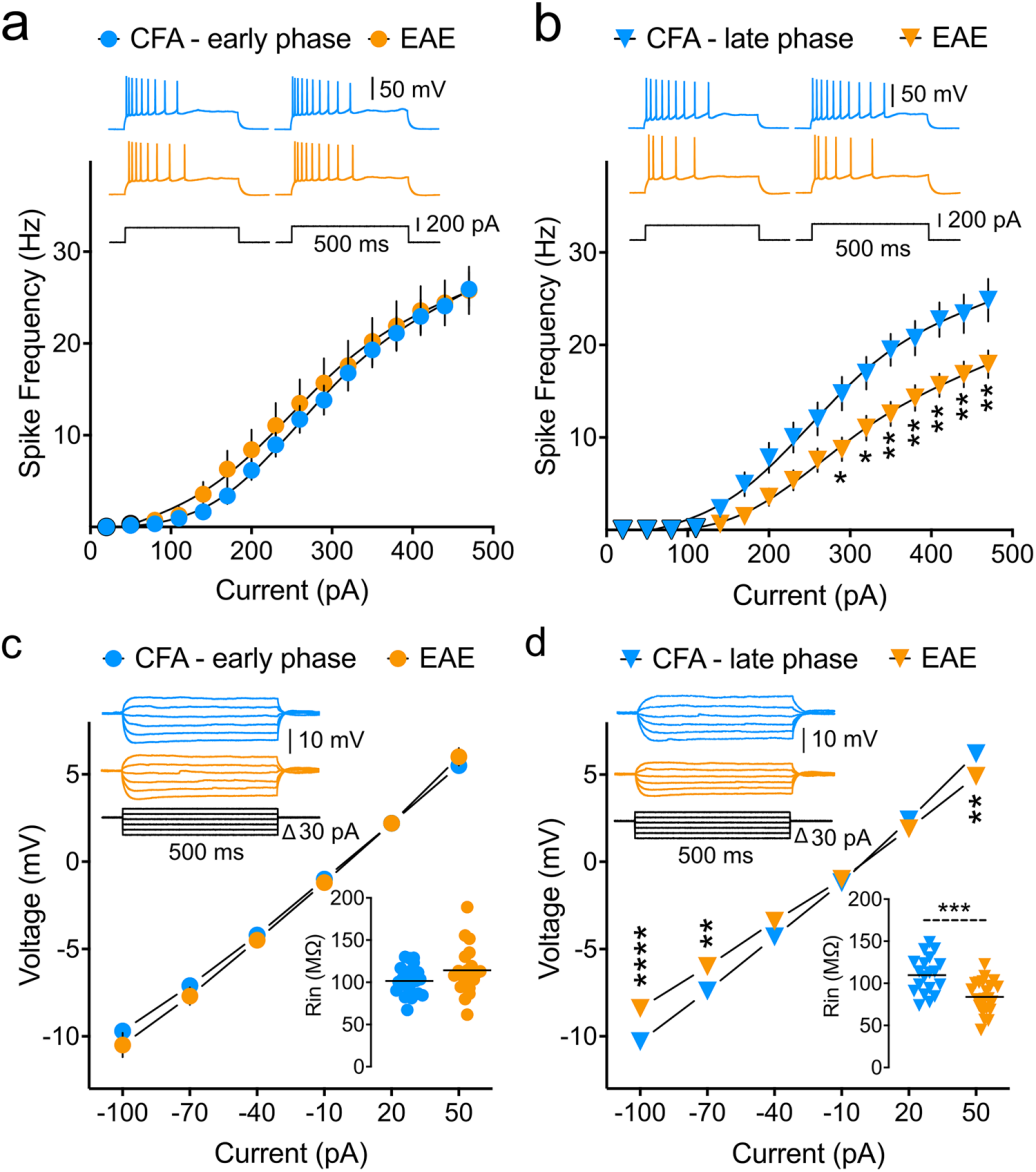
Altered intrinsic excitability of hippocampal CA1 pyramidal neurons in EAE mice. ***a, b***, Representative current-clamp traces (top) illustrate firing responses to step current injections. Spike frequency–current curves showing the relationship between injected current and AP firing rate in hippocampal CA1 pyramidal neurons. ***a***, No significant difference in spike frequency was observed between early CFA-treated (blue circles) and EAE (orange circles) mice. ***b***, Late EAE mice (orange triangles) exhibited a significant reduction in firing rate compared with CFA-treated controls (blue triangles). ***c, d***, Representative voltage traces (top) illustrate membrane responses to current injections. *I*–*V* relationships indicating membrane properties of CA1 pyramidal neurons. ***c***, No significant difference was found between early CFA-treated (blue circles) and EAE (orange circles) mice. ***d***, Late EAE mice (orange triangles) showed a significant decrease in input resistance (Rin) compared with CFA-treated controls (blue triangles). *p < 0.05; **p < 0.01; ***p < 0.001; ****p < 0.0001.

To determine whether the intrinsic properties of hippocampal CA1 pyramidal neurons are affected in EAE, we performed whole-cell patch–clamp recordings in acute brain slices from control (CFA) and EAE mice. Firing frequency of CA1 pyramidal neurons was first evaluated in presymptomatic (early EAE) and symptomatic EAE (late EAE) C57BL/6 mice. Figure 2, *a* and *b*, shows voltage responses and APs in response to the indicated step current injection of CA1 pyramidal neurons of representative control (CFA, blue traces) and early and late EAE (orange traces) mice. When the injected current was increased, the firing rate of symptomatic EAE CA1 pyramidal neurons was significantly different from the CFA group (two-way ANOVA test, *F*_(1,79)_ = 15.72; *p* = 0.0002). Multiple comparisons by Šídák post hoc analyses revealed that the statistically significant effect was attributed to a lower firing rate in CA1 pyramidal neurons of late EAE mice compared with CFA (for all significant values *p* < 0.05; Fig. 2*b*). On the other hand, there were no significant effects on the firing rate of early EAE CA1 pyramidal neurons compared with controls (Fig. 2*a*), indicating decreased excitability only in late EAE mice. Although initial membrane potential has been reported to influence neuronal firing rates in response to current injection, no significant differences in RMP were observed between EAE and control groups (two-way ANOVA, *F*_(1,79)_ = 0.4748; *p* = 0.4928; early, CFA −67.0 ± 0.6 mV; *n* = 23; *N* = 6; EAE −65.6 ± 1.0 mV; *n* = 19; *N* = 4; late, CFA −66.5 ± 0.8 mV; *n* = 19; *N* = 7; EAE −66.8 ± 0.7 mV; *n* = 22; *N* = 7).

Given that RMP was unaltered, other potential factors affecting firing frequency could be input resistance (Rin) and the threshold voltage for AP generation. Therefore, these intrinsic membrane properties in the hippocampal neurons of EAE mice were also investigated. Figure 2, *c* and *d*, shows current–voltage (*I*–*V*) curves constructed by measuring the voltage response of CA1 pyramidal neurons elicited by step current injections from RMP. When comparing changes in Rin, a significantly different phase-dependent effect of EAE was observed in CA1 pyramidal neurons (two-way ANOVA, *F*_(1,79)_ = 5.206; *p* = 0.0252). Multiple comparisons revealed a ∼24% decrease in Rin from CA1 pyramidal neurons of late EAE (CFA 109.7 ± 5.09 ΜΩ; *n* = 19; *N* = 7; EAE 83.85 ± 4.12 ΜΩ; *n* = 22; *N* = 7; *p* = 0.0019; Fig. 2*d*), but not early EAE mice (CFA 101.5 ± 3.55 ΜΩ; *n* = 23; *N* = 6; EAE 114.2 ± 6.68 ΜΩ; *n* = 19; *N* = 4; *p* = 0.2581; Fig. 2*c*), when compared with CFA controls.

**Figure 3.**
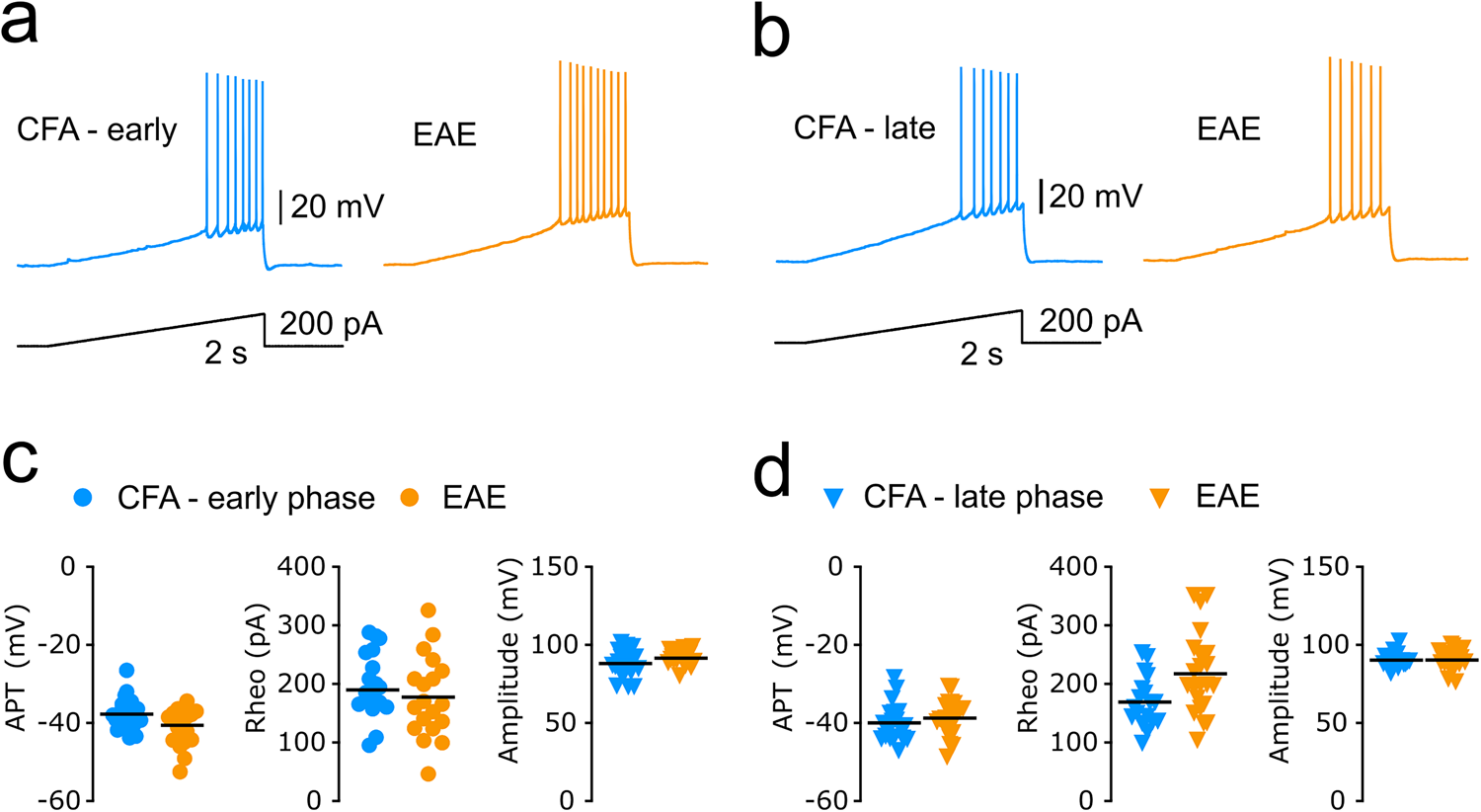
Unaltered AP properties in CA1 pyramidal neurons from EAE mice. ***a, b***, Representative traces of APs evoked by ramp current injections in CA1 pyramidal neurons from CFA-treated (control, blue) and EAE (orange) mice during early (***a***) and late (***b***) disease stages. ***c, d***, Quantification of AP threshold, rheobase, and amplitude shows no significant differences between CFA-treated and EAE mice (*p* > 0.05).

A second factor that could influence neuronal firing frequency is the threshold for AP generation. To assess this, we examined AP properties using ramp current injections in CA1 pyramidal neurons from early and late stages of EAE (Fig. 3*a*,*b*). Representative voltage responses to ramp stimulation are shown for CFA and EAE neurons. Quantification of AP properties revealed no significant differences between groups. During the early phase of EAE, AP threshold was similar between CFA and EAE neurons (CFA, −37.7 ± 0.8 mV; *n* = 23; *N* = 6; EAE, −41.2 ± 1.1 mV; *n* = 19; *N* = 4; Tukey's multiple-comparisons test, *p* = 0.08; Fig. 3*c*). Likewise, rheobase (CFA, 199.02 ± 13.3 pA; EAE, 177.4 ± 15.9 pA; *p* = 0.7) and AP amplitude (CFA, 88.1 ± 1.7 mV; EAE, 91.5 ± 1.1 mV; *p* = 0.29) were not significantly different. Similarly, during the late phase of EAE, AP threshold remained unchanged (CFA, −39.9 ± 1.1 mV; *n* = 19; *N* = 7; EAE, −38.7 ± 0.9 mV; *n* = 22; *N* = 7; *p* = 0.81; Fig. 3*d*). No significant differences were detected in rheobase (CFA, 169.1 ± 10.1 pA; EAE, 219.4 ± 14.9 pA; *p* = 0.07) or AP amplitude (CFA, 90.2 ± 1.0 mV; EAE, 90.3 ± 1.3 mV; *p* = 0.99). Together, these results indicate that the reduced firing frequency observed in CA1 pyramidal neurons during late EAE is unlikely to arise from alterations in AP generation mechanisms. Instead, these findings support the idea that the decreased excitability observed in EAE is likely related to changes in passive membrane properties.

### Basal excitatory and inhibitory synaptic transmission is preserved in CA1 pyramidal neurons during EAE

**Figure 4.**
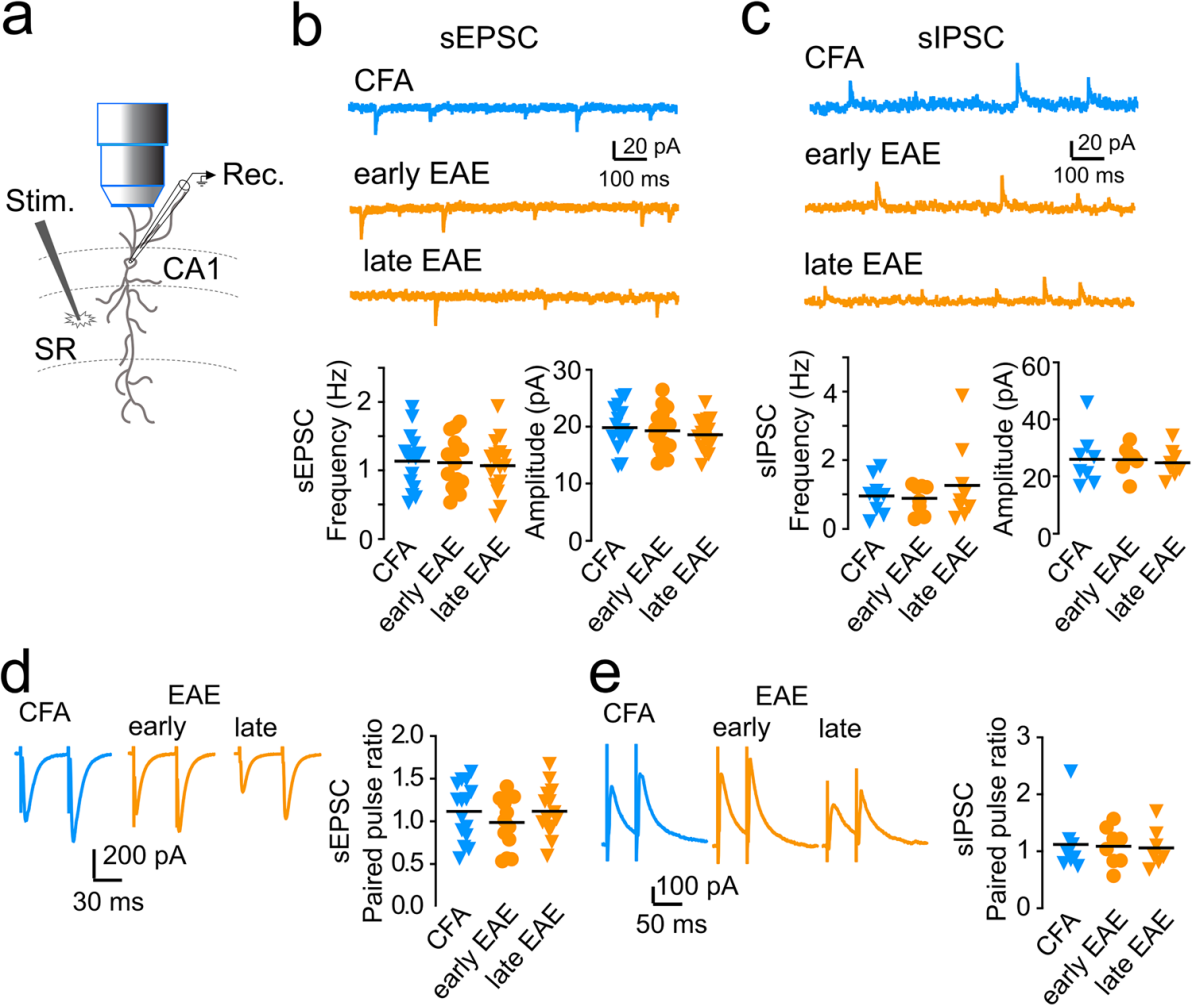
Excitatory and inhibitory synaptic transmission are preserved in CA1 pyramidal neurons during EAE. ***a***, Schematic representation of the hippocampal CA1 region indicating the recording site in pyramidal neurons and the stimulation site in stratum radiatum (SR). ***b***, Representative traces of sEPSCs recorded from CA1 pyramidal neurons in CFA (blue), early EAE (orange), and late EAE (orange) conditions. Bottom, Quantification of sEPSC frequency (left) and amplitude (right) shows no significant differences between groups. ***c***, Representative traces of sIPSCs recorded from CFA, early EAE, and late EAE neurons. Bottom, Summary plots of sIPSC frequency (left) and amplitude (right) reveal no significant changes across conditions. ***d***, Representative traces of paired-pulse eEPSCs in CFA, early EAE, and late EAE groups. Quantification of PPR shows no differences between groups, indicating preserved presynaptic glutamate release probability. ***e***, Representative traces of paired-pulse evoked IPSCs recorded from CA1 pyramidal neurons. PPR of IPSCs is unchanged across CFA, early EAE, and late EAE groups, indicating no detectable alterations in presynaptic GABA release. Statistical comparisons revealed no significant differences between groups (ns).

To determine whether the alterations in intrinsic excitability observed in EAE mice are associated with changes in synaptic input, we examined both excitatory and inhibitory synaptic transmission in hippocampal CA1 pyramidal neurons using whole-cell voltage–clamp recordings. sEPSCs were recorded at a holding potential of −70 mV, and sIPSCs were recorded at 0 mV in acute hippocampal slices obtained from CFA control, early EAE, and late EAE mice (Fig. 4*b*,*c*).

Representative recordings revealed similar spontaneous synaptic activity profiles across groups for both sEPSCs and sIPSCs. Quantitative analysis of sEPSC interevent interval and amplitude distributions showed no significant differences between CFA, early EAE, and late EAE neurons (Fig. 4*b*, sEPSC frequency, CFA, 1.134 ± 0.108 Hz; *n* = 15; *N* = 4; early EAE, 1.112 ± 0.093 Hz; *n* = 16; *N* = 5; late EAE, 1.069 ± 0.099 Hz; *n* = 16; *N* = 5; one-way ANOVA, *F*_(2,44)_ = 0.1109; *p* = 0.895; and amplitude, CFA, 19.83 ± 0.99 pA; early EAE, 19.27 ± 0.93 pA; late EAE, 18.58 ± 0.70 pA; one-way ANOVA, *F*_(2,44)_ = 0.50; *p* = 0.60).

Similarly, analysis of inhibitory synaptic transmission revealed no significant alterations in sIPSC frequency or amplitude between CFA, early EAE, and late EAE groups (Fig. 4*c*, average sIPSC frequency, CFA, 0.9556 ± 0.1772 Hz; *n* = 9; *N* = 4; early EAE, 0.8825 ± 0.1470 Hz; *n* = 8; *N* = 4; late EAE, 1.260 ± 0.3824 Hz; *n* = 9 *N* = 5; one-way ANOVA, *F*_(2,23)_ = 0.5734; *p* = 0.5714; and amplitude, CFA, 25.96 ± 3.319 pA; early EAE, 25.83 ± 1.937 pA; late EAE, 24.74 ± 1.796 pA; one-way ANOVA, *F*_(2,20)_ = 0.07402; *p* = 0.9289), indicating preserved phasic GABAergic input during disease progression.

To assess whether presynaptic release probability was altered, we measured PPRs of evoked excitatory and inhibitory postsynaptic currents by delivering two stimuli separated by a 50 ms interstimulus interval. PPRs of eEPSCs did not differ significantly between groups (CFA, 1.117 ± 0.086; *n* = 15; *N* = 4; early EAE, 0.987 ± 0.085; *n* = 13; *N* = 4; late EAE, 1.119 ± 0.078; *n* = 14; *N* = 4; one-way ANOVA, *F*_(2,39)_ = 0.7881; *p* = 0.46), indicating preserved presynaptic glutamate release probability (Fig. 4*d*). Likewise, PPRs of evoked IPSCs were unchanged across CFA, early EAE, and late EAE neurons (Fig. 4*e*, CFA, 1.121 ± 0.1912; *n* = 8; *N* = 4; early EAE, 1.089 ± 0.1177; *n* = 8; *N* = 4; late EAE, 1.060 ± 0.1315; *n* = 7; *N* = 4; one-way ANOVA, *F*_(2,39)_ = 0.7881; *p* = 0.46), indicating no detectable alterations in presynaptic GABA release probability.

Together, these results demonstrate that basal phasic excitatory and inhibitory synaptic transmissions, as well as presynaptic release properties, are preserved in CA1 pyramidal neurons during EAE progression. These findings indicate that the reduced input resistance and intrinsic excitability observed in late EAE neurons are unlikely to arise from changes in synaptic transmission and instead support an important contribution of intrinsic membrane mechanisms.

### Larger Ih currents are associated with changes in Rin of CA1 neurons in late EAE

We further investigated potential mechanisms underlying the observed reduction in Rin and excitability of CA1 pyramidal neurons during late EAE. Previous studies have demonstrated that hyperpolarization-activated cation currents (Ih), mediated by HCN channels, play a key role in setting resting membrane properties and controlling dendritic excitability (Magee, 1998; Robinson and Siegelbaum, 2003; He et al., 2014). Increased Ih conductance is known to reduce Rin and dampen excitability by providing an inward, depolarizing leak current that stabilizes membrane potential. Therefore, we tested whether Ih currents are upregulated in CA1 pyramidal neurons from EAE mice and could mechanistically explain the phase-dependent decrease in Rin observed during the symptomatic phase. Ih currents were recorded in response to a series of hyperpolarizing voltage steps from a holding potential of −70 to −130 mV in 10 mV increments.

**Figure 5.**
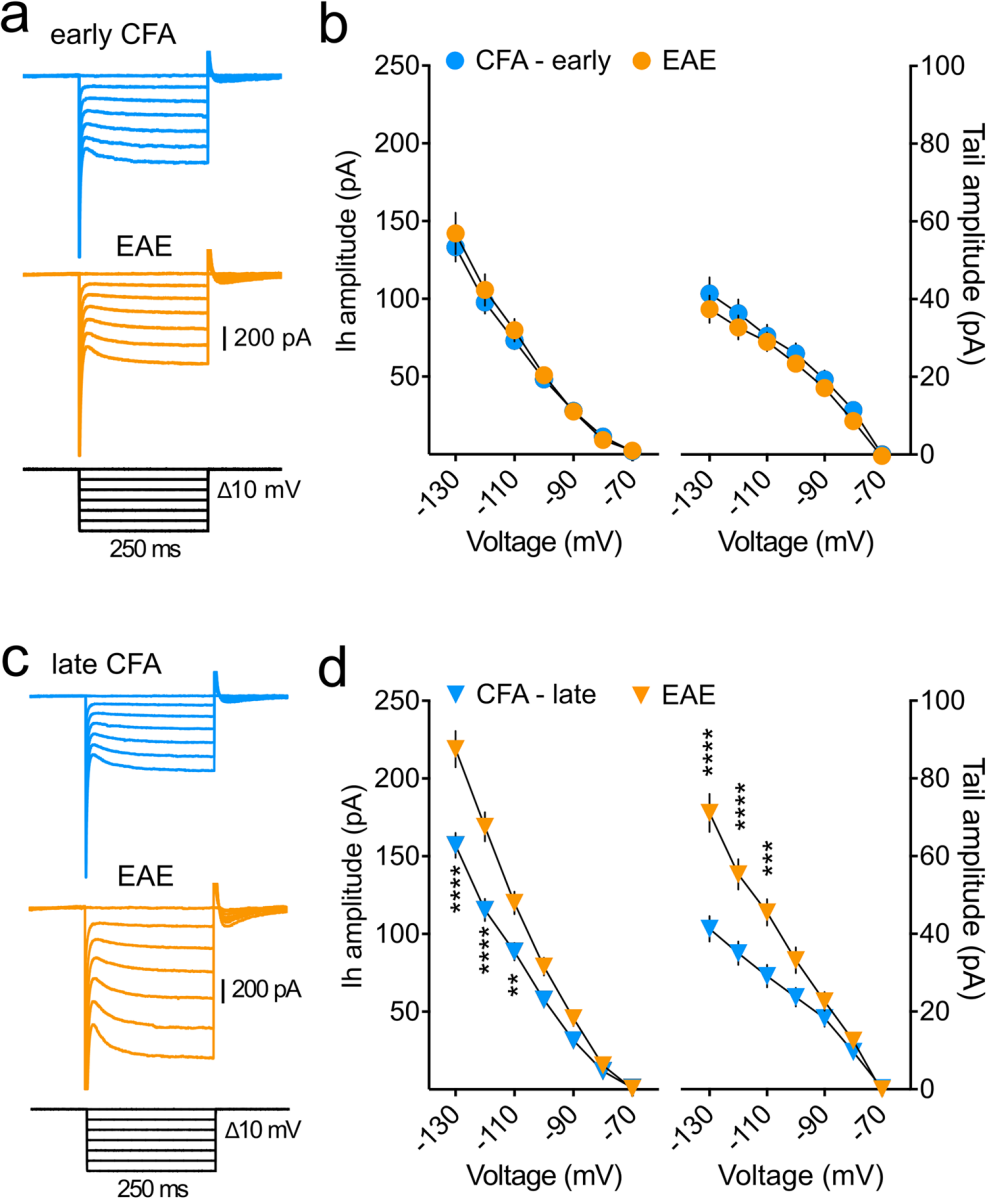
Alterations in Ih current in CA1 pyramidal neurons during EAE progression. ***a, b***, Representative traces (***a***) and quantification (***b***) of Ih and tail current amplitudes show no significant differences in CA1 pyramidal neurons recorded from CFA (blue lines/circles) and early EAE (orange lines/circles) groups. ***c, d***, Representative traces (***c***) and quantification (***d***) of Ih and tail current amplitudes are significantly larger in late EAE (orange lines/triangles) compared with CFA (blue lines/triangles), particularly at hyperpolarized voltages. Data in the *I*–*V* curves are presented as mean ± SEM. **p < 0.01; ***p < 0.001; ****p < 0.0001.

Comparison of current traces and *I*–*V* plots revealed a phase-dependent effect on Ih amplitude of early (Fig. 5*a*,*b*) and late (Fig. 5*c*,*d*) CFA and EAE mice. In early EAE, Ih amplitude and tail currents were not significantly different from CFA controls across the voltage range tested (Fig. 5*b*, left; two-way ANOVA, *F*_(1,259)_ = 1.104; *p* = 0.2944; right, *F*_(1,259)_ = 3.138; *p* = 0.0777). In contrast, CA1 pyramidal neurons from late EAE mice exhibited significantly increased Ih amplitude compared with CFA controls (Fig. 5*c*). Statistical analysis of I–V curves revealed differences between both CFA and EAE groups (Fig. 5*d*, two-way ANOVA, *F*_(1,259)_ = 63.21; *p* < 0.0001), and post hoc comparisons showed significantly larger Ih amplitudes at voltages more negative than −100 mV in late EAE. For instance, a main effect on amplitude was observed at −130 mV (Šídák's multiple-comparisons test, CFA, 157 ± 8 pA; *n* = 19; *N* = 7; EAE, 218.9 ± 11.7 pA; *n* = 20; *N* = 6; *p* < 0.0001; Fig. 5*d*, left). Consistently, tail current analysis also revealed significantly enhanced amplitudes in late EAE (two-way ANOVA *F*_(1,259)_ = 57.88; *p* < 0.0001; Fig. 5*d*, right), with a main effect observed at −130 mV (Šídák's multiple-comparisons test, CFA, 41.3 ± 3.3 pA; *n* = 19; *N* = 7; EAE, 71.1 ± 4.9 pA; *n* = 20; *N* = 6; *p* < 0.0001; Fig. 5*d*, right). These data indicate that Ih current is selectively upregulated in CA1 pyramidal neurons during the symptomatic phase of EAE, but not during early stages, suggesting a potential compensatory or maladaptive mechanism contributing to altered neuronal excitability in late-stage neuroinflammation.

### Increased ZD7288-sensitive Ih current and HCN1 expression in CA1 pyramidal neurons from EAE mice

To determine whether the increased Ih current observed in CA1 pyramidal neurons during EAE contributes to the reduced input resistance and excitability, we used ZD7288, a selective blocker of HCN channels. Unlike the previous voltage step protocol (Fig. 5), here we used a more hyperpolarized holding potential (−40 mV) and stepped to −140 mV in 20 mV increments, optimizing the recruitment of Ih and allowing us to isolate the ZD7288-sensitive component by subtraction.

**Figure 6.**
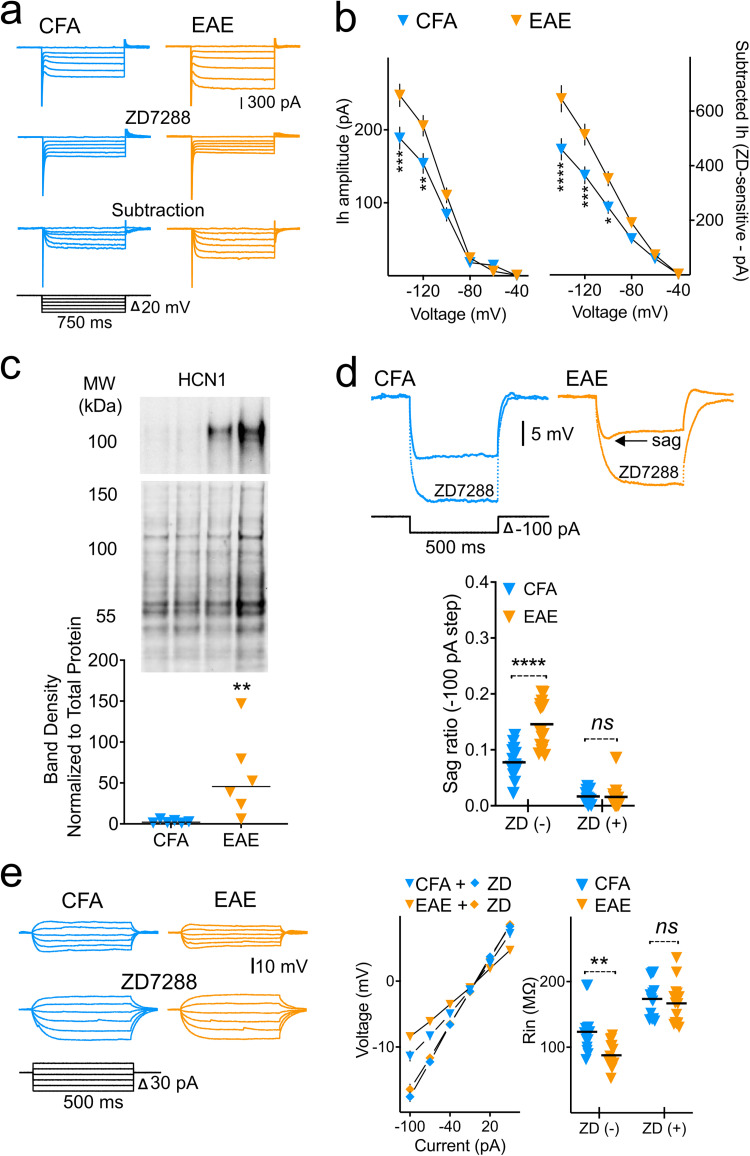
Increased Ih and HCN1 expression in CA1 pyramidal neurons during EAE. ***a***, Representative voltage-clamp traces showing Ih in CA1 pyramidal neurons from CFA (blue) and EAE (orange) groups before (top) and after (middle) bath application of ZD7288, a selective Ih channel blocker. Bottom, Subtraction traces representing ZD7288-sensitive Ih. ***b***, *I*–*V* relationships of total Ih (left) and ZD7288-sensitive Ih (right) show a significant increase in Ih amplitude in EAE neurons compared with CFA. ***c***, Western blot analysis reveals a significant increase in HCN1 protein expression in the hippocampus of EAE mice compared with CFA controls. Representative blot (top) and quantification of band density normalized to total protein (bottom). ***d***, Current-clamp recordings obtained during hyperpolarizing current steps (−100 pA) illustrating increased voltage sag in EAE neurons relative to CFA controls. Application of ZD7288 abolishes sag in both groups. Bottom, Quantification of sag ratio shows a significant increase in late EAE neurons under control conditions that is eliminated by ZD7288. ***e***, Representative current–clamp recordings obtained in CA1 pyramidal neurons from CFA and EAE groups under control conditions and after ZD7288 application. Scatterplot shows a significantly lower input resistance (Rin) in EAE compared with CFA in baseline conditions (*p* < 0.0063), but ZD7288 application largely eliminates this difference (ns, not significant). Data are presented as mean ± SEM. *p < 0.05; **p < 0.01; ***p < 0.001; ****p < 0.0001.

As shown in Figure 6*a*, voltage-clamp recordings revealed larger inward currents in EAE mice, which were strongly attenuated by bath application of ZD7288. Subtraction of the postdrug response from baseline yielded a larger ZD7288-sensitive Ih current in late EAE (orange traces) neurons compared with CFA controls (blue traces). Quantification of amplitude (Fig. 6*b*) confirmed a significant increase in both total Ih (two-way ANOVA, *F*_(1,162)_ = 15.89; *p* = 0.0001) and ZD7288-sensitive Ih (*F*_(1,162)_ = 32.06; *p* < 0.0001), and main effects were observed at −140 mV in EAE neurons (Šídák's multiple-comparisons test, total Ih, CFA, 188.5 ± 15.0 pA; *n* = 14; *N* = 5; EAE, 247.2 ± 15.3 pA; *n* = 15; *N* = 6; *p* = 0.002; Fig. 6*b*, left; and ZD7288-sensitive Ih, CFA, 460.06 ± 38.83 pA; *n* = 14; *N* = 5; EAE, 645.52 ± 48.25 pA; *n* = 15; *N* = 6; *p* < 0.0001; Fig. 6*b*, right, respectively) compared with control CFA mice, consistent with the hypothesis that Ih is functionally upregulated during EAE.

In addition, because HCN1 channels are a major contributor to Ih in CA1 pyramidal neurons, we quantified HCN1 protein levels by Western blot to determine whether functional increases in Ih were accompanied by changes in HCN1 expression. As shown in Figure 6*c*, HCN1 band intensity was significantly elevated in EAE mice compared with CFA (Mann–Whitney test, CFA, 2.72 ± 0.82; *N* = 6; vs EAE, 57.77 ± 20.49; *p* = 0.004; *N* = 6), confirming molecular upregulation of the channel responsible for Ih in CA1 neurons.

To determine whether the functional upregulation of Ih observed in voltage-clamp recordings translated into altered membrane voltage responses, we quantified voltage sag in current-clamp recordings during hyperpolarizing current injections. As shown in Figure 6*d*, CA1 pyramidal neurons from EAE mice exhibited a larger sag in response to a −100 pA current step compared with CFA controls, consistent with enhanced activation of Ih. Quantification of the sag ratio revealed a significant increase in EAE neurons relative to CFA under baseline conditions (two-way ANOVA, *F*_(1,54)_ = 21.77; *p* < 0.0001; Šídák's multiple-comparisons test, CFA, 0.07756 ± 0.00758; *n* = 14; *N* = 5; EAE, 0.14574 ± 0.01081; *n* = 15; *N* = 6; *p* < 0.0001). Importantly, bath application of ZD7288 abolished voltage sag in both groups and eliminated the difference between CFA and EAE neurons (ZD7288-treated, CFA, 0.01664 ± 0.00306; *n* = 14; *N* = 5; EAE, 0.01569 ± 0.00544; *n* = 15; *N* = 6; *p* > 0.9999). These results provide an independent current-clamp confirmation of Ih upregulation in late EAE neurons and support the conclusion that enhanced Ih activation contributes to the increased voltage sag observed in this condition.

Finally, we assessed the impact of ZD7288 on input resistance to evaluate the contribution of Ih conductance to the altered intrinsic properties observed in symptomatic EAE. In current-clamp recordings, application of ZD7288 significantly increased input resistance in EAE neurons, effectively eliminating the difference in Rin between EAE and CFA groups (Fig. 6*e*, two-way ANOVA, *F*_(1,54)_ = 8.32; *p* = 0.0056; Šídák's multiple-comparisons test, CFA, 123.5 ± 9.02 ΜΩ; *n* = 14; *N* = 5; EAE, 87.77 ± 4.91 ΜΩ; *n* = 15; *N* = 6; *p* = 0.0063; and ZD7288-treated, CFA, 173.61 ± 7.23 ΜΩ; EAE, 166.76 ± 7.93 ΜΩ; *p* = 0.83). These data indicate that enhanced Ih current contributes to the reduced input resistance of CA1 pyramidal neurons in EAE and is associated with altered intrinsic excitability.

## Discussion

In this study, we demonstrate that EAE induces significant alterations in the intrinsic excitability of hippocampal CA1 pyramidal neurons. Specifically, we observed a decrease in input resistance (Rin) and a reduction in the AP firing rate, suggesting an overall reduction in excitability. These changes were accompanied by an increase in the hyperpolarization-activated Ih current and elevated expression of HCN1 channels. These findings provide novel insights into how neuroinflammatory conditions such as EAE alter neuronal function and may contribute to hippocampal dysfunction associated with MS.

One of the key findings in this study is that Rin in CA1 pyramidal neurons is reduced in EAE mice. A decrease in Rin suggests an increase in resting membrane conductance, likely driven by enhanced activity of leak channels or specific voltage-gated conductances. Given that we did not observe changes in AP threshold or rheobase, the reduced excitability is likely related primarily with changes in passive membrane properties rather than spike initiation mechanisms. This is consistent with previous reports of altered hippocampal function in neuroinflammatory conditions (Nistico et al., 2013; Di Filippo et al., 2015).

These findings also indicate that the observed reduction in excitability is associated with an increase in Ih current. Ih is mediated by HCN channels and plays a crucial role in setting the RMP and shaping neuronal excitability (Robinson and Siegelbaum, 2003). The elevated Ih observed in EAE mice may contribute to the reduced Rin and excitability, since Ih activation introduces a depolarizing conductance that counteracts hyperpolarization. For instance, acquired epilepsy is associated with region-specific dysregulation of Ih and HCN1 expression, which can either increase or decrease neuronal excitability depending on the pathophysiological context (Poolos et al., 2002; Richichi et al., 2008; Zhao et al., 2023). Similarly, in neuroinflammatory models induced by lipopolysaccharide treatment, CA1 pyramidal neurons exhibit suppressed Ih amplitude alongside concomitant reductions in HCN1 and TRIP8b protein expression (Frigerio et al., 2018). Our findings indicate that Ih upregulation is associated with altered hippocampal intrinsic neuronal function during autoimmune inflammation.

The elevated HCN1 expression observed in EAE mice strengthens the association between Ih upregulation and reduced excitability in CA1 pyramidal neurons. HCN1 is a major subunit contributing to Ih in these neurons and plays an important role in shaping synaptic integration and dendritic excitability (Ludwig et al., 2003; Nolan et al., 2004, 2007). The parallel increase in HCN1 protein and Ih suggests coordinated molecular and functional adaptations. Because CA1 pyramidal neurons express multiple HCN channel subunits, including HCN1 and HCN2, future studies will be needed to determine whether additional HCN isoforms also contribute to the increased Ih observed in EAE.

An important question raised by these findings is what drives the upregulation of Ih and HCN1 expression in CA1 pyramidal neurons during late EAE and what functional role this change serves. Our electrophysiological and pharmacological data support the idea that increased Ih contributes to reduced Rin and altered intrinsic excitability. These observations suggest that Ih upregulation may represent an intrinsic adaptive response associated with neuroinflammatory conditions.

One plausible mechanism underlying this change is activity-dependent homeostatic intrinsic plasticity. Several studies have reported increased network excitability in models of demyelination. In the cuprizone model, chronic demyelination is associated with spontaneous hippocampal seizures and epileptiform activity (Hoffmann et al., 2008). Additionally, demyelination disrupts inhibitory circuitry through the loss of parvalbumin-positive interneurons and complement-mediated elimination of inhibitory synapses, resulting in reduced feedforward inhibition within hippocampal networks (Ramaglia et al., 2021). More recent work demonstrates progressive hippocampal seizure activity, glutamate dysregulation, and structural remodeling of pyramidal neurons during chronic demyelination (Anderson et al., 2025). These findings indicate that demyelinating pathology can shift hippocampal circuits toward hyperexcitability. Although such network alterations were not directly assessed in the present study, these observations provide a potential conceptual framework through which inflammatory or demyelinating conditions could influence intrinsic membrane adaptations. Within this context, the upregulation of Ih observed here may represent a compensatory intrinsic response associated with altered network states during neuroinflammatory disease.

EAE is characterized by persistent neuroinflammation, and proinflammatory cytokines such as TNF-α and IL-1β have been shown to modulate intrinsic neuronal excitability and voltage-gated ion channels through signaling pathways including NF-κB, MAPK, and cAMP-dependent mechanisms (Viviani et al., 2003; Santello and Volterra, 2012). Moreover, HCN channels are highly sensitive to intracellular signaling cascades, including cAMP-dependent mechanisms that are altered under inflammatory conditions (Biel et al., 2009). Therefore, Ih upregulation may reflect the combined influence of inflammatory signaling and activity-dependent homeostatic processes during neuroinflammatory disease progression.

Upregulation of Ih has been shown to act as a negative feedback mechanism, countering excessive hyperpolarization and dampening excitability in various disease models (Richichi et al., 2008). HCN channel expression and Ih currents are known to be dynamically regulated by neuronal activity, and activity-dependent increases in Ih have been described in hyperexcitable states such as epilepsy (Poolos et al., 2002). Thus, enhanced Ih conductance may represent an adaptive response that limits excessive excitability, although potentially at the cost of altered synaptic integration.

Because Ih critically regulates theta-frequency resonance and synaptic plasticity, its upregulation could influence hippocampal-dependent memory and cognitive function (Stark et al., 2013). HCN channels regulate temporal summation and backpropagation of APs (Magee, 1998; Nolan et al., 2004), and their dysregulation could impair synaptic timing and information processing.

Importantly, we did not observe significant changes in spontaneous excitatory or inhibitory synaptic transmission in EAE mice across disease stages, indicating that the reduced excitability observed is unlikely to be explained solely by alterations in basal synaptic input but instead is associated with changes in intrinsic membrane properties. Previous studies have reported alterations in hippocampal inhibitory circuits during EAE (Ziehn et al., 2010; Kammel et al., 2018), and impaired long-term potentiation (Di Filippo et al., 2013), demonstrating synaptic dysfunction in other contexts. Although tonic GABAergic conductances were not assessed, our data indicate that phasic synaptic transmission onto CA1 pyramidal neurons remains largely preserved, supporting the important role of intrinsic membrane mechanisms in the altered excitability observed during disease progression.

Because Ih strongly influences dendritic integration and synaptic potential dynamics in CA1 pyramidal neurons, including EPSP amplitude and temporal summation, it will be important to determine whether its upregulation in EAE alters these properties. Although our recordings focused on basal synaptic transmission and presynaptic release probability, they did not directly assess EPSP integration. Addressing this will be essential to define how Ih-dependent intrinsic changes may influence hippocampal circuit function.

The functional implications of these findings warrant further investigation. The observed decrease in excitability may represent an adaptive response associated with limiting excessive neuronal firing in the presence of neuroinflammation. Alternatively, it may contribute to hippocampal dysfunction associated with MS, as reduced excitability of hippocampal neurons could influence information processing and memory encoding. Future studies should explore whether pharmacological modulation of HCN channels can alter these physiological changes and potentially influence cognitive function in EAE models.

It is interesting that our previous studies revealed altered synaptic transmission in the amygdala during early EAE (Acharjee et al., 2018), supporting region-specific effects of neuroinflammation. Similarly, Ih regulation differs across demyelination models. For example, in the cuprizone model, Ih is reduced in thalamic relay neurons alongside decreased HCN channels expression (Chaudhary et al., 2022), in contrast to the Ih upregulation observed here. These differences likely reflect distinct pathological mechanisms, as cuprizone induces toxin-mediated oligodendrocyte loss, whereas EAE is driven by autoimmune inflammation (Torkildsen et al., 2008; Ransohoff, 2012). Together, these findings indicate that Ih regulation and intrinsic excitability may be strongly context-dependent across brain regions and disease models.

These findings suggest that modulation of HCN1 channels and Ih currents could influence neuronal function in MS. While current therapies target demyelination and inflammation, intrinsic neuronal excitability represents a complementary physiological process that may contribute to disease-related dysfunction. Preclinical suppression of HCN1 activity, via shRNA knockdown in dorsal CA1 (Kim et al., 2012) or pharmacological inhibition (Pinares-Garcia et al., 2023), increases neuronal excitability and produces antidepressant-like behavioral effects. Conversely, corticosterone-induced stress elevates HCN1 expression and Ih in the dorsal hippocampus (Kim et al., 2022), indicating that this pathway can be maladaptively upregulated. Collectively, these findings support the idea that restoring Ih balance may represent a potential strategy to modulate hippocampal dysfunction in neuroinflammatory conditions.

In conclusion, our study provides evidence that EAE alters the intrinsic excitability of CA1 pyramidal neurons and is associated with increased Ih currents and elevated HCN1 expression. These findings suggest that HCN channel modulation may represent a potential therapeutic target for improving hippocampal dysfunction in neuroinflammatory diseases such as MS. Understanding the cellular and molecular mechanisms underlying these changes will be critical for developing targeted interventions to preserve cognitive function in patients affected by neuroinflammatory disorders.

## Synthesis

Reviewing Editor: Arianna Maffei, Stony Brook University Decisions are customarily a result of the Reviewing Editor and the peer reviewers coming together and discussing their recommendations until a consensus is reached. When revisions are invited, a fact-based synthesis statement explaining their decision and outlining what is needed to prepare a revision will be listed below. The following reviewer(s) agreed to reveal their identity: NONE. Note: If this manuscript was transferred from JNeurosci and a decision was made to accept the manuscript without peer review, a synthesis may not be available.

The revised manuscript addressed the remaining concerns. The authors have toned down speculations and clarified remaining issues. I do not have additional concerns
